# Onasemnogene Abeparvovec in Type I Spinal Muscular Atrophy: 24‐Month Follow‐Up From the Italian Registry

**DOI:** 10.1002/acn3.70356

**Published:** 2026-03-19

**Authors:** Marika Pane, Giorgia Coratti, Chiara Cutrì, Antonio Varone, Riccardo Masson, Adele D'Amico, Valeria Sansone, Sonia Messina, Federica Ricci, Chiara Ticci, Claudio Bruno, Caterina Agosto, Francesca Benedetti, Antonella Pini, Sabrina Siliquini, Massimiliano Filosto, Alberto Zambon, Ilaria Bitetti, Maria Rosaria Manna, Claudia Dosi, Riccardo Zanin, Stefano Parravicini, Roberto De Sanctis, Giulia Stanca, Michela Catteruccia, Michele Tosi, Irene Mizzoni, Emilio Albamonte, Valentina Franchino, Maria Sframeli, Ilaria Cavallina, Elena Procopio, Michele Sacchini, Simone Morando, Noemi Brolatti, Federica Trucco, Gaia Scarpini, Elena Briganti, Beatrice Berti, Concetta Palermo, Daniela Leone, Stefano C. Previtali, Eugenio Mercuri, the ITASMAC working group

**Affiliations:** ^1^ Nemo Clinical Center, Fondazione Policlinico Universitario Agostino Gemelli IRCSS Rome Italy; ^2^ Pediatric Neurology, Catholic University of the Sacred Heart Rome Italy; ^3^ Pediatric Neurology, Santobono‐Pausilipon Children's Hospital, AORN Naples Italy; ^4^ Developmental Neurology Unit, Fondazione IRCCS Istituto Neurologico Carlo Besta Milan Italy; ^5^ Unit of Muscular and Neurodegenerative Disorders, Bambino Gesù Children's Hospital, IRCCS Rome Italy; ^6^ The NEMO Clinical Center in Milan, Neurorehabilitation Unit, University of Milan, ASST Niguarda Hospital Milan Italy; ^7^ Unit of Neurodegenerative Diseases, Department of Clinical and Experimental Medicine, University of Messina Messina Italy; ^8^ Child Neuropsychiatry Unit, Children's Hospital Regina Margherita, Department of Public Health and Paediatric Sciences, University of Turin Turin Italy; ^9^ Metabolic and Neuromuscular Unit, Meyer Children's Hospital IRCCS Florence Italy; ^10^ Centre of Translational and Experimental Myology, IRCCS Istituto Giannina Gaslini Genoa Italy; ^11^ Department of Neuroscience, Rehabilitation, Ophthalmology, Genetics, Maternal and Child Health‐DINOGMI, University of Genoa Genoa Italy; ^12^ Department of Women and Children's Health, University of Padova Padova Italy; ^13^ Pediatric Neuromuscular Unit, IRCCS Istituto Delle Scienze Neurologiche di Bologna Bologna Italy; ^14^ Child Neuropsychiatry Unit, Paediatric Hospital G Salesi Ancona Italy; ^15^ Department of Clinical and Experimental Sciences, NeMO‐Brescia Clinical Center for Neuromuscular Diseases, University of Brescia Brescia Italy; ^16^ Neuromuscular Repair Unit, Institute of Experimental Neurology (InSpe), Division of Neuroscience, IRCCS Ospedale San Raffaele Milan Italy; ^17^ Paediatric Neurology and Muscle Disease Unit, Department of Neuroscience, Rehabilitation, Ophthalmology, Genetics, Maternal and Child Health‐DINOGMI, IRCCS Istituto Giannina Gaslini, University of Genoa Genoa Italy; ^18^ Vita‐Salute San Raffaele University Milan Italy

**Keywords:** clinical outcomes, gene therapy, motor function, onasemnogene abeparvovec, spinal muscular atrophy

## Abstract

**Objective:**

Onasemnogene abeparvovec (OA) is an AAV9‐based gene therapy for spinal muscular atrophy type I (SMA I). Real‐world outcomes show increased response variability compared to clinical trials, and follow‐up data beyond 12–18 months are limited. The aim of this 24‐month prospective observational study is to comprehensively describe the clinical outcomes of an Italian cohort of SMA I patients treated with OA.

**Methods:**

Based on recent literature, patients' treatment status was categorized as: monotherapy (OA only), bridge therapy (transition to OA within 3 months of starting nusinersen or risdiplam), or switch therapy (transition to OA after > 3 months of 1st treatment). Linear mixed‐effects models examined predictors of improvement (CHOP‐INTEND), adjusting for baseline motor function, SMN2 copy number, age, and prior treatment. Descriptive analyses were used to show changes in motor, respiratory, and nutritional milestones.

**Results:**

The cohort included 64 patients: 27 monotherapy, 9 bridge, and 28 switch. All patients showed significant improvement over 24 months (*β* = 20.40 points/year, *p* < 0.001). Patients who switched showed slower improvement (*β* = −3.76, *p* = 0.038) compared to monotherapy, while those who bridged showed no difference. Older age at treatment was associated with slower improvement (*β* = −1.48 points/year per month, *p* = 0.002). Of 49 non‐sitters at baseline, 39 (80%) achieved sitting and 5 (10%) achieved walking. No new safety signals emerged in the second year of follow‐up.

**Interpretation:**

Age and baseline motor functional status significantly influence outcomes; however, substantial confounding, particularly the initial treatment, limits the ability to isolate individual effects. Longer follow‐up is essential for evaluating therapeutic responses in heterogeneous SMA I populations.

## 1 Introduction

Spinal muscular atrophy (SMA) is caused by mutations of the survival motor neuron (*SMN*1) gene, resulting in a reduction of functional *SMN* protein that primarily affects spinal motor neurons [1, 2, 3]. This results in progressive muscle weakness that affects skeletal muscle and often respiratory and bulbar function. Historically, SMA was classified according to age at onset and maximum motor functional level achieved [4]. Type I was characterized by onset during the first 6 months of life, inability to achieve unsupported sitting, and was nearly invariably associated with death or the need for permanent ventilation by the end of 20 months [4]. Improvements in standards of care and the advent of disease‐modifying therapies (DMTs) have dramatically improved survival and overall function [5, 6, 7, 8, 9]. Several studies have reported the efficacy of onasemnogene abeparvovec (OA), an adeno‐associated viral (AAV9) vector‐based gene therapy, which introduces a functional copy of the *SMN*1 gene into motor neurons by means of a single intravenous injection [10, 11, 12, 13, 14]. Both the results of the clinical trials, including the long‐term follow‐up, and the real‐world data in naïve patients show a dramatic increase in survival (between 90% and 100%) and a significant functional improvement. The initial studies SMART and STR1VE, performed in selected cohorts of infants below 6 months of age and without bulbar or respiratory complications, reported a quite homogenous pattern of responses, with increased survival and most infants achieving the ability to sit unsupported by the age of 18 months [15, 16]. In contrast, the STR1VE EU study, which also included a number of infants with respiratory and bulbar impairment, showed a more variable clinical response to treatment. A higher number of infants did not achieve independent sitting by the end of the study, suggesting greater variability in outcomes that reflected the motor functional status and the extent of bulbar and respiratory involvement at the time of dosing [16]. This has been further explored in the real‐world data after OA became commercially available [17, 18, 19, 20, 21]. As the approved label for OA also included infants older than 6 months, with no or very limited restrictions on the clinical phenotype in most countries, this has allowed to assess the efficacy of OA in a much wider cohort, also including infants previously treated with another DMT.

Real‐world data with longer follow up confirmed the variability suggested by STR1VE‐EU showing that while there was always a positive response, with most infants surviving and improving on the motor functional scales, not all the infants achieved sitting [16, 17, 19, 22, 23]. Older and heavier infants were at higher risk of a more limited response [19, 20, 24, 25]. In a number of these cases, functional motor improvement was achieved after the age of 18 months thus suggesting that a longer follow‐up may provide better information on the possible achievement of motor milestones [17, 26, 27]. Only a few studies however reported longer follow‐up in patients treated with OA, with the larger ones mainly focusing on infants identified by neonatal screening [17, 18, 19, 21, 22].

Following on our preliminary 1 year results [20], in this study we report our experience in a cohort of 64 type I patients treated with OA for at least 2 years trying to establish trajectories of progression and the possible effect of several variables, such as age, baseline value, or previous treatment.

## 2 Methods

This observational cohort study, following on our preliminary study on 1 year results [20], included symptomatic patients with 5q spinal muscular atrophy (type I) who received onasemnogene abeparvovec (OA) and completed at least 2 years of post‐treatment follow‐up. Data were prospectively collected using the structured electronic CRF of the Italian Spinal Muscular Atrophy Consortium (ITASMAC) Registry [28]. The registry was approved by the institutional Ethics Committee (2355/18 ID: 1894), all parents signed consent forms, and the protocol grants the collection and the analysis of the data for the study of disease trajectories.

The study population included all consecutive SMA type I patients treated with OA at ITASMAc centers who had completed at least 24 months of follow‐up. All patients meeting these criteria were enrolled, irrespective of SMN2 copy number, age at OA initiation, weight at OA initiation, previous DMTs received, or sex.

The cohort included patients with varying pretreatment histories, categorized into three groups: monotherapy (OA only), bridge therapy (transition to OA within 3 months of starting nusinersen or risdiplam), or switch therapy (transition to OA after more than 3 months of nusinersen or risdiplam treatment) [29].

Comprehensive demographic and clinical data were collected at baseline (*at the initiation of OA*), including sex assigned at birth, age and weight at OA dosing, SMN2 copy number, and motor functional status. Motor functional status was classified at the time of OA dosing into three categories: non‐sitter, sitter, or walker [5, 30].

For patients with prior treatment exposure, baseline characteristics were also recorded at the time of initial disease‐modifying therapy initiation, including age at first DMT exposure, CHOP‐INTEND scores on the Children's Hospital of Philadelphia Infant Test of Neuromuscular Disorders (CHOP‐INTEND) [31, 32], and motor functional status. Outcome measures were selected based on patient age and motor functional status according to established SMA care standards [30]. All assessments were performed by evaluators trained and certified in the respective motor scales within the ITASMAC network.

Assessments were conducted at baseline and longitudinally over the 24‐month follow‐up period.

Motor function was assessed using the CHOP‐INTEND, a validated 64‐point scale for evaluating motor abilities in infants with neuromuscular disorders. For a subset of patients demonstrating sufficient motor ability, the Hammersmith Functional Motor Scale Expanded (HFMSE) [33] was administered at baseline and follow‐up visits. For patients under 2 years of age, clinical evaluators scored the HFMSE through play‐based observation rather than formal testing procedures.

Motor milestone acquisition was documented prospectively during clinical visits, with specific attention to achievement of independent sitting and walking, as well as any loss of previously attained milestones. The timing of milestone achievement was categorized as occurring within the physiological window for age‐typical development (on time sitting), outside this window (delayed sitting), or never achieved within the two‐year observation period.

Nutritional status was classified as oral feeding or tube/percutaneous endoscopic gastrostomy feeding at baseline and monitored throughout follow‐up for any transitions between feeding modalities. Respiratory status was similarly documented, with patients being classified as requiring or not requiring non‐invasive (NIV) or invasive ventilatory support. The ventilatory support per day was recorded and stratified by hours per day.

### 2.1 Statistical Analysis

Descriptive statistics are presented as means with standard deviations, medians with interquartile ranges or ranges as appropriate, and frequencies with percentages for categorical variables.

Statistical analyses were conducted to examine differences in baseline characteristics and treatment outcomes across pretreatment groups. Chi‐squared tests were employed to compare age at OA initiation and baseline CHOP‐INTEND scores between treatment groups. Post hoc pairwise comparisons were performed using Wilcoxon rank‐sum tests with Bonferroni correction to control for multiple comparisons.

Linear mixed‐effects models with random intercepts for individual patients were constructed to examine longitudinal changes in CHOP‐INTEND scores over the 24‐month period. To address potential confounding between age at treatment initiation and prior treatment status, two separate multivariable models were developed. The pre‐treatment model included time, baseline CHOP‐INTEND score, SMN2 copy number, pretreatment category, and the interaction between time and pretreatment status. The age model substituted continuous age at OA initiation for categorical pretreatment status, including time, baseline CHOP‐INTEND score, SMN2 copy number, age at initiation, and the interaction between time and age. Both models incorporated interaction terms between time and baseline CHOP‐INTEND to account for ceiling effects, whereby patients with higher baseline motor function demonstrated slower rates of improvement.

Model fit was evaluated using marginal and conditional *R*‐squared values, intraclass correlation coefficients, and residual variance components.

Fisher's exact test compared the proportion of patients achieving sitting across pretreatment groups. Kruskal–Wallis test assessed differences in age at sitting achievement.

Patients were excluded if they had received OA for < 24 months at the time of last follow‐up. Among the included cohort meeting the 24‐month follow‐up requirement, data completeness was 100% for all primary and secondary outcome measures, baseline characteristics, and covariates included in the statistical models, precluding the need for missing data imputation strategies. All statistical tests were two‐sided, and significance was set at *p* < 0.05.

## 3 Results

The cohort included 64 with SMA type I who had at least 2 years of follow‐up after treatment with OA. Prior to OA, 28 patients received nusinersen or risdiplam for more than 3 months [29]. Additionally, 9 patients received a bridging treatment (8 with nusinersen and 1 with risdiplam, for < 3 months [29]).

Table 1 shows demographic and clinical characteristics of the patients included in the final cohort.

**TABLE 1:** Demographic and clinical characteristics of the patients included in the final cohort.

		SMA I (*n* = 64)
*SMN*2 copy number (*N*, %)	2	55 (85.9%)
	3	9 (14.1%)
Sex assigned at birth (*N*, %)	Female	24 (37.5%)
	Male	40 (62.5%)
Age at OA dosing (years)	Mean (SD)	1.52 (1.48)
	Median [Min, Max]	0.770 [0.0548, 4.92]
Weight at OA dosing (*N*, %)	≤ 8.5 kg	37 (57.8%)
	> 8.5 kg	27 (42.2%)
CHOP INTEND score at OA dosing	Mean (SD)	38.0 (13.7)
	Median [Min, Max]	41.0 [4.00, 61.0]
Functional status at OA dosing	Non‐sitter	49 (76.6%)
	Sitter	15 (23.4%)
	Walker	0 (0%)
Treated with a previous DMT	Monotherapy	27 (42.2%)
	Bridge	9 (14.1%) Median duration [Min, Max]: 0.15 [0.00, 0.18]
	Switch	28 (43.8%) Median duration [Min, Max]: 1.96 [0.51, 4.73]
Age at previous DMT dosing (Mean (SD); Median [Range])	Switched	0.59 (0.43) 0.51 [2.37–0.02]
	Bridge	0.63 (0.45) 0.53 [0.07–1.48]
CHOP INTEND score at previous treatment dosing (Mean (SD); Median [Range])	Switched	27.17 (8.79) 29 [9–41]
	Bridge	23.85 (10.1) 25 [7–39]
Functional status at previous treatment dosing	Non sitters	37 (100%)

*Age at OA initiation* differed significantly across pre‐treatment groups, *χ* ^2^(2) = 46.87, *p* < 0.001. Patients who received OA as monotherapy (*n* = 27) received OA at a mean age of 0.33 years (SD = 0.23, Media*n* = 0.27, IQR = 0.15–0.46). Bridge therapy patients (*n* = 9) received OA at 0.72 years (SD = 0.42, Median = 0.47, IQR = 0.41–0.96), while patients who switched to OA (*n* = 28) were significantly older at 2.92 years (SD = 1.16, Median = 3.05, IQR = 2.14–3.87).

*Baseline CHOP‐INTEND scores* differed significantly across pre‐treatment groups, *χ* ^2^(2) = 24.2, *p* < 0.001.

Patients who received OA as monotherapy (*n* = 27) had a mean baseline score of 30.6 (SD = 14.1, Median = 34.0, IQR = 17.5–41). Bridge therapy patients (*n* = 9) had intermediate scores of 40.3 (SD = 11.8, Median = 42, IQR = 32–49), while patients who switched to OA (*n* = 28) had the highest baseline motor function at 47.8 (SD = 6.07, Median = 47.5, IQR = 45–52). Post hoc pairwise comparisons using Wilcoxon rank‐sum tests with Bonferroni correction revealed that patients who switched to OA had significantly higher baseline CHOP‐INTEND scores than patients who received OA as monotherapy (*p* < 0.001). Bridge therapy patients did not differ significantly from either patients who received OA as monotherapy (*p* = 0.18) or patients who switched to OA (*p* = 0.20).

### 3.1 CHOP‐INTEND Results Over 24 Months After Treatment

**FIGURE 1:**
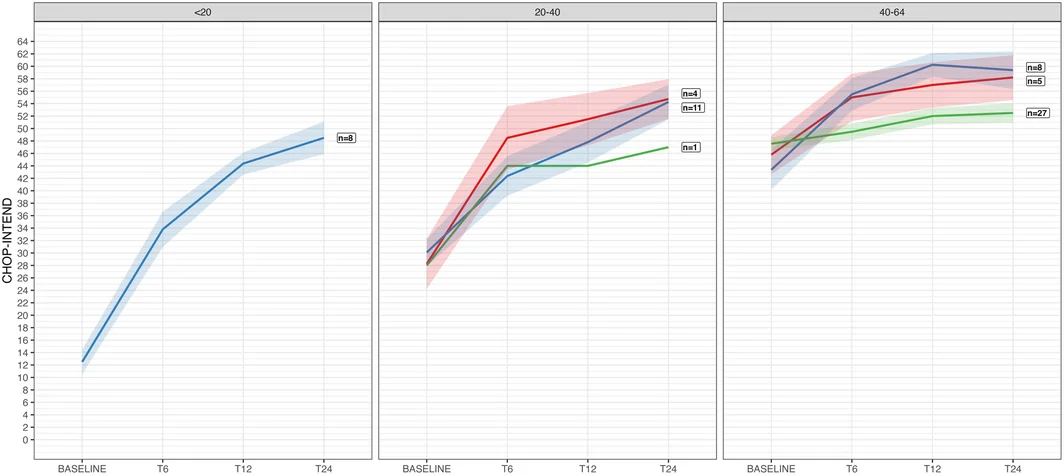
CHOP‐INTEND trajectories over 24 months from OA treatment initiation stratified by baseline CHOP INTEND group, pre‐treatment status. Solid lines represent the estimated mean CHOP‐INTEND scores, with shaded ribbons indicating 95% confidence intervals. Green lines represent patients who switched to OA, Blue represents bridge patients, while red lines denote patients who did not switch to OA.

Figure 1 shows trajectories over 24 months stratified by CHOP‐INTEND at OA dosing group, pretreatment status.

Linear mixed‐effects models with random intercepts for SMA I patients were used to examine changes in CHOP‐INTEND scores over time. To address potential confounding between age at OA initiation and prior treatment status, we examined two multivariable linear mixed models adjusting for baseline CHOP‐INTEND, *SMN*2 copy number, and baseline CHOP‐INTEND (Table 2).

**TABLE 2:** Linear mixed‐effects model with random intercept for patient results.

	Pre‐treatment model			Age model		
	Estimates	CI	*p*	Estimates	CI	*p*
**Predictors**						
(Intercept)	8.19	4.34 to 12.05	< 0.001	8.29	4.58 to 12.05	< 0.001
Time	20.40	16.06 to 24.74	< 0.001	20.67	16.61 to 24.73	< 0.001
Baseline CHOP‐INTEND	0.83	0.72 to 0.95	< 0.001	0.84	0.74 to 0.94	< 0.001
Bridge (< 2 months) vs. Monotherapy	1.18	−2.44 to 4.80	0.523			
Long‐term (≥ 2 months) vs. Monotherapy	−0.20	−3.43 to 3.04	0.904			
*SMN2* copy number (3 vs. 2)	2.97	−0.09 to 6.04	0.057	3.24	0.43 to 6.05	0.024
Time × Baseline CHOP‐INTEND	−0.29	−0.42 to −0.17	< 0.001	−0.29	−0.40 to −0.18	< 0.001
Time × Bridge	−1.00	−5.21 to 3.21	0.640			
Time × Long‐term pre‐treatment	−3.76	−7.30 to −0.22	0.038			
Age at OA initiation				−0.18	−1.09 to 0.73	0.702
Time × Age at OA initiation				−1.48	−2.46 to −0.50	0.002
**Random effects**						
*σ* ^2^	37.88			37.73		
*τ*_11_	12.21_ID*time_			10.05_ID*time_		
ICC	0.30			0.26		
*N*	64_ID_			64_ID_		
Observations	249			249		
Marginal *R* ^2^/Conditional *R* ^2^	0.631/0.741			0.653/0.741		

In the pre‐treatment model (Model 1), patients demonstrated significant improvement over time (*β* = 20.40 points/year, 95% CI: 16.06–24.74, *p* < 0.001). After adjusting for baseline CHOP‐INTEND scores and *SMN*2 copy number, patients who switched to OA showed significantly slower improvement compared to patients who received OA as monotherapy (Time × Long‐term interaction: *β* = −3.76, 95% CI: −7.30 to −0.22, *p* = 0.038). Patients receiving bridge therapy demonstrated similar improvement rates to patients who received OA as monotherapy (Time × Bridge: *β* = −1.00, 95% CI: −5.21 to 3.21, *p* = 0.640).

The age model (Model 2) revealed that older age at OA initiation was associated with significantly slower improvement (Time × Age: *β* = −1.48 points/year per month of age, 95% CI: −2.46 to −0.50, *p* = 0.002), though the main effect of age at baseline was not significant (*β* = −0.18, *p* = 0.702).

Both models demonstrated a significant ceiling effect (Time × Baseline CHOP: *β* ≈ −0.29, *p* < 0.001), indicating that patients with higher baseline scores improved more slowly.

*SMN*2 copy number was associated with better outcomes, reaching significance in the age model (*β* = 3.24, *p* = 0.024) and showing a trend in the pre‐treatment model (*β* = 2.97, *p* = 0.057).

### 3.2 Change in Functional, Nutritional and Respiratory Status

Fifteen patients were already sitters at OA initiation; of these, 1 lost sitting position for severe scoliosis and 1 achieved walking.

One out of 49 non‐sitters at OA initiation achieved sitting position but lost it soon after due to motor function decline.

Forty out of 49 non‐sitter at OA initiation achieved sitting position (80%), while 5 achieved independent walking (10%).

#### 3.2.1 Age at OA Initiation

Of the 40 non‐sitter at OA initiation who later achieved sitting position, 20 were treated before 6 months of age (10 achieved sitting within the physiological window of 6–12 months), 11 were treated between 6 and 12 months of age (1 achieved sitting within the physiological window), and 9 were treated after the age of 12 months (none achieved sitting within the physiological window). Among the 5 patients who achieved walking, all were treated before 6 months of age (4 achieved walking within the physiological window of 12–18 months).

#### 3.2.2 Time From OA to Achieving Sitting

Of the 40 non‐sitter at OA initiation who later achieved sitting position, 16 (40%) achieved it within 6 months after OA initiation, 13 (32.5%) within 12 months, 6 (15%) within 18 months and 5 beyond 18 months (12.5%). Of the cohort of patients treated before 6 months of age, all patients with 3*SMN2* copy number achieved sitting on‐time (within 6 to 12 months from OA treatment).

Table S1 presents data on timing of sitting achievements after treatment with OA for patients who started treatment before 6 months of age subdivided by SMN2 copy number and timing from OA initiation.

**FIGURE 2:**
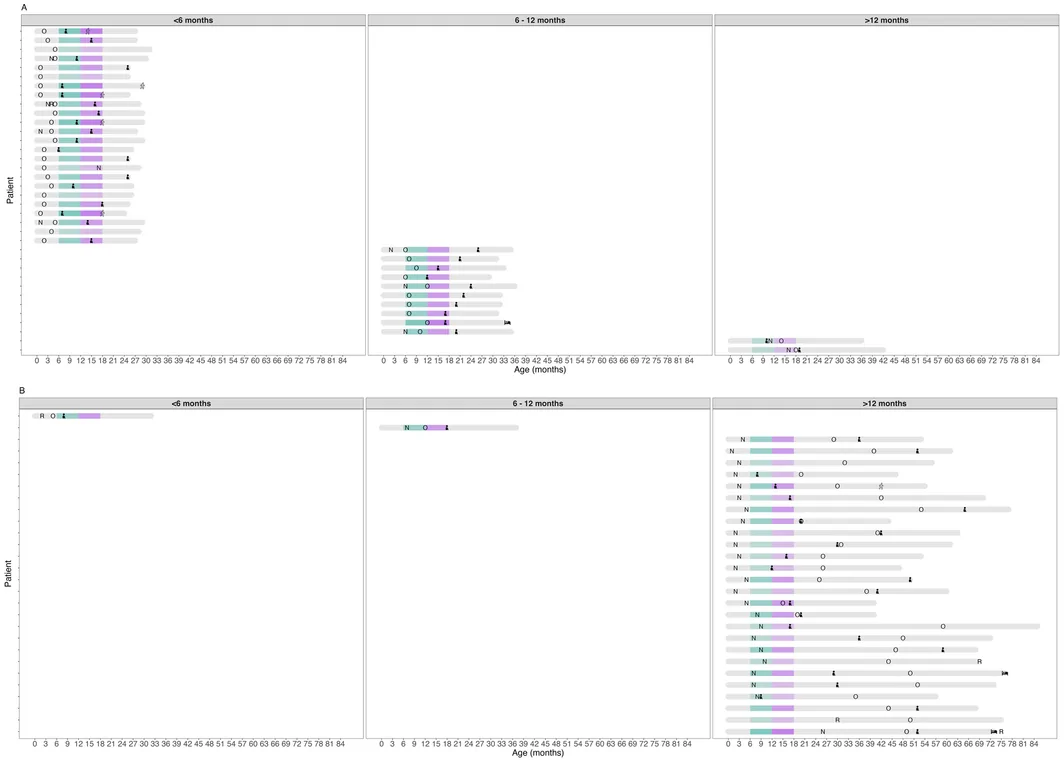
Individual motor milestones acquisition by age at OA treatment and by treatment status. Panel A: Naïve & Bridge patients, Panel B: Patients who switched to OA. *N* = nusinersen initiation, *R* = risdiplam initiation, O = onasemnogene abeparvovec initiation, green shadowing = window of physiological sitting acquisition, purple shadowing = Window of physiological walking acquisition, sitting acquisition = sitting icon, Walking acquisition = walking icon, Lost sitting position = bed icon.

Figure 2 shows individual trajectories of motor milestone acquisitions (sitting, walking) subdivided by age at OA treatment and treatment status.

### 3.3 Monotherapy

Of the 27 monotherapy patients, 5 never achieved sitting position (all treated before 6 months of age), while 22 achieved sitting after OA initiation.

#### 3.3.1 Age at OA Initiation

Of the 22 who achieved sitting after OA initiation, 15 were treated before 6 months of age (8 achieved sitting within the physiological window of 6–12 months) and 7 were treated between 6 and 12 months of age (1 achieved sitting within the physiological window). Among the 5 patients who achieved walking (defined as walking independently > 10 m), all were treated before 6 months of age (4 achieved walking within the physiological window of 12–18 months).

#### 3.3.2 Time From OA to Achieving Sitting

Of the 22 who achieved sitting after OA initiation, 10 (45.5%) achieved it within 6 months after OA initiation, 4 (18.2%) within 12 months, 5 (22.7%) within 18 months and 3 (13.6%) beyond 18 months.

### 3.4 Bridge to OA

Of the 9 who bridged to OA, 2 achieved sitting before OA initiation, while 7 achieved sitting after OA initiation.

#### 3.4.1 Age at OA Initiation

Of the 7 patients who achieved sitting after OA initiation, 4 were treated before 6 months of age (1 achieved sitting within the physiological window of 6–12 months) and 3 were treated between 6 and 12 months of age (0 achieved sitting within the physiological window).

#### 3.4.2 Time From OA to Achieving Sitting

Of the 7 patients who achieved sitting after OA initiation, 1 (14.3%) achieved it within 6 months after OA initiation, 5 (71.4%) within 12 months and 1 (14.3%) beyond 18 months.

### 3.5 Switched to OA

Of the 28 who switched to OA, 13 achieved sitting before OA initiation, while 11 achieved sitting after OA initiation.

#### 3.5.1 Age at OA Initiation

Of the 11 patients who achieved sitting after OA initiation, 1 was treated before 6 months of age (achieved sitting within the physiological window of 6–12 months), 1 was treated between 6 and 12 months of age (did not achieve sitting within the physiological window), and 9 were treated after 12 months of age (therefore all achieved sitting outside the physiological window).

#### 3.5.2 Time From OA to Achieving Sitting

Of the 11 patients who achieved sitting after OA initiation, 5 (45.4%) achieved it within 6 months after OA initiation, 4 (36.4%) within 12 months, 1 (9.1%) within 18 months, and 1 (9.1%) beyond 18 months.

The proportion of individuals achieving sitting did not differ significantly across treatment groups (*p* = 0.45), as their time to sitting achievement (*p* = 0.48).

Table 3 stratifies these results by treatment category (monotherapy, bridge, switched to OA) and motor functional status at OA initiation.

**TABLE 3:** Change in functional status.

OA initiation status → outcome	Monotherapy (*n* = 27)	Bridge (*n* = 9)	Switched to OA (*n* = 28)
**Non‐sitter at OA initiation**	***n* = 27**	***n* = 7**	***n* = 15**
Remained non‐sitter	5 (22%)	0 (0%)	4 (27%)
→ Achieved sitting	22 (78%)	7 (100%)	11 (73%)
→ Achieved standing	6 (22%)	2 (29%)	1 (7%)
→ Achieved walking	5 (19%)	0 (0%)	0 (0%)
**Sitter at OA initiation**	***n* = 0**	***n* = 2**	***n* = 13**
Remained sitter	—	2 (100%)	12 (92%)
→ Lost sitting			1 (8%)
→ Achieved standing	—	0 (0%)	2 (15%)
→ Achieved walking	—	0 (0%)	1 (8%)

Details of baseline distribution of *SMN2* copy number, CHOP‐INTEND, nutritional and ventilatory status of patients who achieved the sitting position within the physiological window per age (on time), outside the physiological window per age (delayed sitting) or never achieved the sitting position within the 2 years of follow‐up are shown in Table 4.

**TABLE 4:** Distribution of SMN2 copy number, CHOP INTEND, nutritional and ventilatory status at OA initiation of patients who achieved the sitting position within the physiological window per age (on time), outside the physiological window per age (delayed sitting) or never achieved the sitting position within the 2 years of follow‐up.

Characteristics	OA initiated < 6 months			OA initiated 6–12 months		OA initiated > 12 months	
	Sitting never acquired, *N* = 5	Delayed sitting, *N* = 10	On time sitting, *N* = 9	Delayed sitting, *N* = 9	On time sitting, *N* = 1	Delayed sitting, *N* = 1	On time sitting, *N* = 1
Nutritional status at OA initiation							
Oral	5 (100%)	8 (80%)	8 (89%)	9 (100%)	1 (100%)	1 (100%)	1 (100%)
Tube	0 (0%)	2 (20%)	1 (11%)	0 (0%)	0 (0%)	0 (0%)	0 (0%)
Ventilatory status at OA initiation							
Non‐invasive ventilation	4 (80%)	6 (60%)	2 (22%)	3 (33%)	1 (100%)	0 (0%)	1 (100%)
No respiratory support	1 (20%)	4 (40%)	7 (78%)	6 (67%)	0 (0%)	1 (100%)	0 (0%)
*SMN*2 copy number							
2	5 (100%)	10 (100%)	5 (56%)	7 (78%)	0 (0%)	0 (0%)	0 (0%)
3	0 (0%)	0 (0%)	4 (44%)	2 (22%)	1 (100%)	1 (100%)	1 (100%)
CHOP‐INTEND at OA initiation							
< 20	3 (60%)	3 (30%)	0 (0%)	2 (22%)	0 (0%)	0 (0%)	0 (0%)
20–40	2 (40%)	6 (60%)	2 (22%)	3 (33%)	1 (100%)	0 (0%)	1 (100%)
40–64	0 (0%)	1 (10%)	7 (78%)	4 (44%)	0 (0%)	1 (100%)	0 (0%)

*Note:* Numbers are *n* (%). Statistical testing not performed due to small sample sizes in some strata and perfect separation.

#### 3.5.3 Nutritional Aspects

Ten patients had already a tube inserted at OA initiation; of these, 2 switched to oral feeding over follow‐up. Of the 54 already oral fed at OA initiation, 3 had a tube inserted over follow‐up (2 during the 1st year and 1 during the 2nd year of follow‐up). Table 5 stratifies these results by treatment category (monotherapy, bridge, switched to OA) and nutritional status at OA initiation.

**TABLE 5:** Change in nutritional and ventilatory status.

	Monotherapy (*n* = 27)	Bridge (*n* = 9)	Switched to OA (*n* = 28)
**Nutritional status**			
At OA initiation			
Oral feeding	24 (88%)	9 (100%)	21 (75%)
Tube/PEG feeding	3 (11%)	0 (0%)	7 (25%)
Deterioration during FU			
Oral → PEG placement	2 (8%)	0 (0%)	1 (5%)
Improvement during FU			
Tube/PEG → Oral feeding	2 (67%)	—	0 (0%)
**Respiratory status**			
At OA initiation			
No respiratory support	16 (59%)	3 (33%)	2 (7%)
Respiratory support	11 (41%)	6 (67%)	26 (93%)
Deterioration during FU			
Spontaneous → Support needed	7 (44%)	0 (0%)	1 (50%)
New tracheostomy placement	0 (0%)	0 (0%)	0 (0%)
Improvement during FU			
Support → No respiratory support	1 (9%)	1 (17%)	1 (4%)
Hours of ventilation at 24 months			
> 8 h/day	3 (16%)	2 (40%)	9 (36%)
6–8 h/day	9 (47%)	1 (20%)	12 (48%)
≤ 5 h/day	4 (21%)	2 (40%)	4 (16%)
As needed	3 (16%)	0 (0%)	0 (0%)

#### 3.5.4 Respiratory Status

Forty‐three patients already required ventilatory support at baseline, of these, none required a tracheostomy and 3 were weaned off ventilation over follow‐up. Of the 21 who did not require respiratory support at OA initiation, 8 required ventilatory support over follow‐up (5 during the 1st year and 3 during the 2nd year of follow‐up). Table 4 stratifies these results by treatment category (monotherapy, bridge, switched to OA) and respiratory status at OA initiation.

Data S1 report details a subgroup of patients who demonstrated sufficient motor ability to complete portions of the HFMSE assessment.

### 3.6 Safety

We conducted systematic safety surveillance throughout the second year following OA administration. This analysis identified no adverse events. First‐year safety data, including acute and subacute adverse events, have been previously reported in our initial cohort analysis [20].

## 4 Discussion

Our results, obtained in a cohort of 64 patients with SMA I followed for at least 24 months after treatment with OA, provided a longer follow up can help to better establish both efficacy and long‐term safety. Although OA has now been available for several years, the number of studies reporting efficacy and safety beyond the first 12 months after treatment is scanty. Most of the available studies have a limited number of individuals with follow up longer than 12 months and include both symptomatic and asymptomatic infants [17, 18, 21].

In this paper we focused on evaluating efficacy and safety 24 months after treatment with OA on symptomatic patients only, also accounting for factors that may affect the timing of the clinical responses. While the importance of early intervention is well established [12, 13, 15, 16, 34] we have also explored other variables that were previously found to affect the response to OA. Both clinical studies and real‐world data have demonstrated some of the infants with respiratory and bulbar difficulties at time of treatment with OA do not always achieve the ability to sit unsupported within 1 year after treatment or by the age of 18 months [12, 13, 15, 16, 34].

As suggested by some of the previous studies assessing OA efficacy, the analysis and the interpretation of clinical changes in real world cohorts can be challenging as when OA became available may individuals had previously been treated with another disease‐modifying therapy [8, 35, 36]. Further challenges also come from the elevated number of other clinical and genetic variables in children with SMA I. The analysis of our cohort confirmed that all these variables contribute, to different extents, to the variability of outcome observed over a long‐term follow up. Our analysis showed a strong correlation between age at OA initiation and prior treatment status (*p* < 0.001). Patients who switched to OA were significantly older at OA initiation compared to patients who received OA as monotherapy, creating substantial confounding between these two predictors. Additionally, patients who switched to OA had markedly higher baseline CHOP‐INTEND scores compared to patients who received OA as monotherapy (mean: 47.8 ± 6.07 vs. 30.6 ± 14.1, *p* < 0.001). These findings—older age, higher baseline motor function, and slower improvement—represent a complex interpretative challenge. Our models explicitly controlled for baseline CHOP‐INTEND scores and included a Time × Baseline CHOP‐INTEND interaction term to account for the well‐established ceiling effect of this scale. The observed slower improvement in patients who switched to OA (*β* = −4.15, *p* = 0.021) represents an effect beyond their higher starting scores and cannot be attributed solely to ceiling constraints. Their higher baseline scores reflect treatment benefit from initial therapy, but their slower subsequent improvement may indicate they had already captured much of their potential therapeutic gain or represent cases where disease progression is more gradual.

Given the strong correlation between age and pre‐treatment status, definitively separating their independent contributions is not possible with this observational dataset. Indeed, when modeled separately, both factors significantly predicted slower improvement (pre‐treatment: *p* = 0.021; age: *p* = 0.002). This pattern suggests that pre‐treatment status may capture age‐related variance more comprehensively than chronological age alone, possibly because treatment history serves as a proxy for disease duration, phenotypic severity, and other factors that influence therapeutic response.

In the younger group, the numbers were too small and the cohort too heterogeneous to allow a meaningful clinical analysis stratified for different variables, but we tried to identify possible patterns that could be more predictive of acquiring milestones such as sitting. The infants treated within the age of 6 months were mainly treatment naïve at the time they received OA except for a few (*n* = 9) who received a short bridging therapy with nusinersen (*n* = 8) or risdiplam (*n* = 1).

It is of interest that even within this relatively more homogeneous subgroup the outcome at the 2nd year post treatment was variable. In this early treated group, all the infants with 3*SMN*2 copies were able to sit unsupported within 18 months of follow up. In contrast, those with 2 *SMN*2 copies had a variable outcome, ranging from sitting within the physiological window per age to never being able to achieve sitting within the timeframe of 2 years of follow‐up. Low CHOP‐INTEND scores at time of treatment, and/or the presence of bulbar and respiratory difficulties were more often found to be associated to a less favorable, but this did not always hold true for individual cases.

When other aspects were considered, of the infants who had no obvious respiratory or bulbar impairment at the time of treatment, < 5% (3/64) required a gastrostomy (2 during the 1st year and 1 during the 2nd year of follow‐up) and 12% required non‐invasive ventilation (8/64) (5 during the 1st year and 3 during the 2nd year of follow‐up); of these, only 2 individuals needed both gastrostomy and non‐invasive ventilation.

Interestingly, in agreement with previous reports, there was no new safety signal between the first and the second year.

In conclusion, our study supports the hypothesis that there is continuous improvement over the second year after treatment and that important milestones, such as the ability to sit unsupported, may be achieved after the first year of treatment. Notably, < 20% (9/49) of the patients who were unable to sit at treatment initiation did not achieve the ability to sit within the 2 years of observation.

These findings are clinically relevant when considered against the natural history of SMA type I as, by definition, untreated patients never achieve independent sitting and invariably experience progressive motor decline leading to death or permanent ventilation by the age of 2 years in over 90% of the cases [37, 38, 39]. Achieving independent sitting represents a transformative functional milestone that enhances independence in daily activities, facilitates social interaction, and reduces caregiver burden.

Some of the variability in outcome or in the onset of milestones can be explained by age or baseline motor function, but the strong confounding among age and pre‐treatment status does not allow us to definitively attribute the slower improvement solely to one of these factors.

Our analysis focused specifically on motor trajectories following OA administration rather than examining complete longitudinal data from initial diagnosis through all treatment transitions, as a valid comparison of outcomes across these groups presents methodological challenges. Patients with different treatment histories also differ in age, disease severity, and clinical characteristics. To account for these differences—including confounding by indication and selection bias—larger cohorts and more advanced methods such as propensity score matching would be required. Despite these limitations, our study addresses clinically relevant questions regarding what factors predict motor outcomes after OA treatment in real‐world settings where most patients receive some form of disease‐modifying therapy before potential OA administration. Future studies in larger cohorts may help to better address other functional aspects, such as cognitive and behavioral or speech and language profiles, to fully characterize the long‐term neurodevelopmental trajectory of OA‐treated patients.

## Author Contributions

M.P. and G.C. conceptualization, study design, writing – original draft, and project administration; G.C. formal analysis and statistical methodology; Clinical assessments, data collection, data management, project administration and/or outcome measure administration were performed by C.C., A.V., R.M., A.D., V.S., S.M., F.R., C.T., C.B., C.A., F.B., A.P., S.S., M.F., A.Z., I.B., M.R.M., C.D., R.Z., S.P., R.D.S., G.S., M.C., M.T., I.M., E.A., V.F., M.S., I.C., E.P., M.S., S.M., N.B., F.T., G.S., E.B., B.B., C.P., D.L. and S.C.P.; All authors contributed to writing – review and editing and approved the final version of the manuscript; E.M. conceptualization, supervision, funding acquisition, and writing – review and editing.

## Funding

The study was partly funded by the Piano Nazionale di Ripresa e Resilienza—PNRR finanziato dall'Unione europea—NextGenerationEU—Missione 4 “Istruzione e ricerca”—Componente 2 “Dalla ricerca all'impresa”—Investimento 1.1 “Fondo per il Programma Nazionale di Ricerca e Progetti di Rilevante Interesse Nazionale (PRIN)” Progetto PRIN 2022 Spinal muscular atrophy: clinical phenotypes and biomarkers at the time of the new disease modifying therapies—Prot. 2022N2FHA8—CUP J53D23011020008. Dr. Coratti is partially supported by the Italian Ministry of Health (GR‐2021‐12374579), Dr. Masson is partially supported by the Italian Ministry of Health (RRC). All authors are part of ERN Euro‐NMD. The Italian registry is partially funded by Biogen, Novartis, and Roche. Funders had no role in the study design; in the collection, analysis, and interpretation of data; in the writing of the report; and in the decision to submit the paper for publication.

## Conflicts of Interest

Prof. Marika Pane: Speaking honoraria and advisory board participation from Roche, Novartis, Biogen, Scholar Rock, Pfizer, PTC, Santhera, Italfarmaco; not related to this manuscript. Dr. Giorgia Coratti: Speaking honoraria and advisory board participation from Roche, Novartis, Biogen, Scholar Rock, Avexis; not related to this manuscript. Dr. Riccardo Masson: Advisory board and speaker honoraria from Novartis, Roche, Biogen, Pfizer, PTC, Santhera, Italfarmaco; not related to this manuscript. Dr. Adele D'Amico: Speaking honoraria and advisory board participation from Roche, Novartis; not related to this manuscript. Prof. Valeria Sansone: Speaking honoraria and advisory board participation from Roche, Novartis, Biogen; not related to this manuscript. Prof. Sonia Messina: Speaking honoraria and advisory board participation from Roche, Novartis, Biogen, Genzyme; not related to this manuscript. Prof. Federica Ricci: Advisory board participation for Santhera; speaking honoraria from Roche, Novartis, Biogen, Sanofi‐Genzyme; not related to this manuscript. Dr. Chiara Ticci: Speaking honoraria and advisory board participation from Roche, Novartis, Biogen; not related to this manuscript. Dr. Claudio Bruno: Speaking honoraria and advisory board participation from Roche, Novartis, Biogen; not related to this manuscript. Dr. Antonella Pini: Advisory board participation for Biogen; principal investigator in clinical trials for Biohaven; teaching initiatives for PTC, Biogen, Roche; not related to this manuscript. Dr. Massimiliano Filosto: Advisory board participation for Biogen; teaching initiatives for Biogen, Roche; consulting fees for Biogen; not related to this manuscript. Dr. Roberto De Sanctis: Speaking honoraria from Roche, Novartis, Biogen, Avexis; not related to this manuscript. Dr. Emilio Albamonte: Advisory board participation for Roche, Novartis, Biogen, PTC; Speaking honoraria from Roche, Novartis, Biogen, PTC; not related to this manuscript. Dr. Federica Trucco: Participation in scientific advisory boards for Roche UK and Italy; teaching initiatives for Biogen, Avexis, Roche, BREAS; not related to this manuscript. Dr. Concetta Palermo: Teaching initiatives for Biogen; not related to this manuscript. Prof. Stefano C. Previtali: Advisory board participation for Argenx, Alexion, Wave, Takeda, Santhera, Esperare; principal investigator in clinical trials for Argenx, Alexion, AstraZeneca, Wave, Dyne, Esperare, Takeda, Kedrion, Avidity, Dianthus, Immunovant, ImmunoAbs, Entrada, Sanofi, Vertex; speaking honoraria from Argenx, Takeda; not related to this manuscript. Prof. Eugenio Mercuri: Speaking honoraria and advisory board participation from Roche, Novartis, Biogen, Scholar Rock, Pfizer, PTC, Santhera, Italfarmaco, Solid; not related to this manuscript. The other authors declare no conflicts of interest.

## Supporting information


**Appendix S1:** acn370356‐sup‐0001‐AppendixS1.docx.

## Data Availability

The data that support the findings of this study are available from the corresponding author upon reasonable request.
