# Supplementary material for: Onasemnogene Abeparvovec in Type I Spinal Muscular Atrophy: 24‐Month Follow‐Up From the Italian Registry
Source: Ann Clin Transl Neurol. 2026 Mar 19;13(9):1866–77. doi: 10.1002/acn3.70356 (PMC13394819; doi:10.1002/acn3.70356)
Supplement: Supplementary file 1 [file ACN3-13-1866-s001.docx]

***ITASMAC working group:***

| **Full name** | **Initials** | **Affiliation** |
| --- | --- | --- |
| Sophia Paolucci | SP | Centro Clinico Nemo, Fondazione Policlinico Universitario Agostino Gemelli IRCCS, Rome, Italy |
| Lavinia Fanelli | LF | Centro Clinico Nemo, Fondazione Policlinico Universitario Agostino Gemelli IRCCS, Rome, Italy |
| Antonella Longo | AL | Unit of Muscular and Neurodegenerative Disorders, Bambino Gesù Children's Hospital, IRCCS, Rome, Italy |
| Nicola Forcina | NF | Centro Clinico Nemo, Fondazione Policlinico Universitario Agostino Gemelli IRCCS, Rome, Italy |
| Giulia Norcia | GN | Centro Clinico Nemo, Fondazione Policlinico Universitario Agostino Gemelli IRCCS, Rome, Italy |
| Sara Carnicella | SC | Centro Clinico Nemo, Fondazione Policlinico Universitario Agostino Gemelli IRCCS, Rome, Italy |
| Maria Teresa Arnoldi | MTA | Developmental Neurology Unit, Fondazione IRCCS Istituto Neurologico Carlo Besta, Milan, Italy |
| Giacomo De Luca | GDL | Unit of Muscular and Neurodegenerative Disorders, Bambino Gesù Children's Hospital, IRCCS, Rome, Italy |
| Irene Giacompolli | IG | The NEMO Clinical Center in Milan, Neurorehabilitation Unit, University of Milan, ASST Niguarda Hospital, Milan, Italy |
| Eloisa Gitto | EG | Unit of Neurodegenerative Diseases, Department of Clinical and Experimental Medicine, University of Messina, 98122 Messina, Italy |
| Roberto Materia | RM | Unit of Neurodegenerative Diseases, Department of Clinical and Experimental Medicine, University of Messina, 98122 Messina, Italy |
| Enrica Rolle | ER | Centro per le Malattie. Neuromuscolari “P. Peirolo” Azienda Ospedaliera Universitaria S. Giovanni Battista di Torino, Torino, Italy |
| Matteo Pirin | MP | Metabolic and Neuromuscular Unit, Meyer Children's Hospital IRCCS, Florence, Italy |
| Chiara Panicucci | CP | Centre of Translational and Experimental Myology, IRCCS Istituto Giannina Gaslini, Genoa, Italy |
| Chiara Bravetti | CB | Centro Clinico Nemo, Fondazione Policlinico Universitario Agostino Gemelli IRCCS, Rome, Italy |
| Stefano Scarparo | SS | Neuromuscular Repair Unit, Institute of Experimental Neurology (InSpe), Division of Neuroscience, IRCCS Ospedale San Raffaele, Milan, Italy |
| Anna Capasso | AC | Centro Clinico Nemo, Fondazione Policlinico Universitario Agostino Gemelli IRCCS, Rome, Italy |
| Gianpaolo Cicala | GC | Centro Clinico Nemo, Fondazione Policlinico Universitario Agostino Gemelli IRCCS, Rome, Italy |
| Marianna Villa | MV | Centro Clinico Nemo, Fondazione Policlinico Universitario Agostino Gemelli IRCCS, Rome, Italy |
| Chiara Arpaia | CA | Centro Clinico Nemo, Fondazione Policlinico Universitario Agostino Gemelli IRCCS, Rome, Italy |
| Gloria Pomè | GP | Developmental Neurology Unit, Fondazione IRCCS Istituto Neurologico Carlo Besta, Milan, Italy |

**Table S1**

|  |  | **Within 6 months from treatment** | **Within 12 months from treatment** | **Within 18 months from treatment** | **After 18 months from treatment** | **Never** |
| --- | --- | --- | --- | --- | --- | --- |
| **3 SMN2 copy number** | *On time sitting* | △ △ △ | □ |  |  | *-* |
|  | *Delayed sitting* |  |  |  |  | *-* |
| **2 SMN2 copy number** | *On time sitting* | △△◧*▲*△ |  |  |  | - |
|  | *Delayed sitting* |  | ◭◧◧◧□ | ◧□ | ●◑○ | *-* |
|  | *Never* |  |  |  |  | *□*◧◐◐◐ |

**Table S1. Details of timing of sitting acquisition since OA initiation for patients treated with OA before 6 months of age.** Legend: Circle= CHOP-INTEND <20, Square= CHOP-INTEND 20-40, Triangle= CHOP INTEND 40-64, half-left filling (◐,◧, ◭)= non-invasive ventilation, half right filling (◑,◨, ◮)= tube feeding, full filling (●,■,▲)= non-invasive ventilation and tube feeding. On time=sitting within 12 moth of age; delayed sitting= sitting after 12 months of age.

**Data S1. Details of a subgroup of patients who demonstrated sufficient motor ability to complete portions of the HFMSE assessment**.

A subset of patients demonstrated sufficient motor ability to complete portions of the HFMSE assessment. For patients under 2 years of age, clinical evaluators scored the assessment through play-based observation; these scores should be interpreted with caution. Table presents HFMSE score data stratified by treatment category, visit type, and SMA type.

|  |  | **SMA I** | | |
| --- | --- | --- | --- | --- |
|  | **HFMSE** | **Monotherapy**  **(n=27)** | **Bridge**  **(n=9)** | **Switched to OA**  **(n=27)** |
| **Baseline** | N (%) | 0 (0%) | 0 (0%) | 5 (19%) |
|  | Mean age (SD) | N/A | N/A | 3.14 (1.15) |
|  | Median age [Min, Max] | N/A | N/A | 2.92 [1.69, 4.39] |
|  | Mean HFMSE (SD) | N/A | N/A | 16.8 (12.3) |
|  | Median HFMSE [Min, Max] | N/A | N/A | 16.0 [7.00, 37.0] |
| **6 months** | N (%) | 1 (4%) | 1 (11%) | 7 (26%) |
|  | Mean age (SD) | 0.89 () | 1.93 () | 3.18 (1.11) |
|  | Median age [Min, Max] | 0.89 () | 1.93 () | 3.36 [1.82, 4.85] |
|  | Mean HFMSE (SD) | 22 () | 2 () | 12.4 (5.68) |
|  | Median HFMSE [Min, Max] | 22 () | 2 () | 13.0 [4.00, 19.0] |
| **12 months** | N (%) | 5 (19%) | 3 (33%) | 12 (44%) |
|  | Mean age (SD) | 1.21 (0.263) | 1.95 (0.455) | 3.45 (1.15) |
|  | Median age [Min, Max] | 1.13 [0.978, 1.56] | 1.95 [1.50, 2.41] | 3.31 [1.32, 5.27] |
|  | Mean HFMSE (SD) | 26.6 (20.8) | 16.7 (9.02) | 17.7 (12.2) |
|  | Median HFMSE [Min, Max] | 27.0 [6.00, 56.0] | 16.0 [8.00, 26.0] | 15.0 [5.00, 45.0] |
| **24 months** | N (%) | 9 (%) | 3 (%) | 17 (%) |
|  | Mean age (SD) | 2.28 (0.206) | 2.94 (0.567) | 4.67 (1.04) |
|  | Median age [Min, Max] | 2.27 [2.03, 2.68] | 2.92 [2.38, 3.52] | 4.48 [2.64, 6.32] |
|  | Mean HFMSE (SD) | 34.1 (19.1) | 16.3 (6.35) | 19.1 (11.0) |
|  | Median HFMSE [Min, Max] | 29.0 [5.00, 66.0] | 20.0 [9.00, 20.0] | 17.0 [5.00, 45.0] |

**HFMSE scores subdivided by treatment category and SMA type**.
